# A new psychosocial goal-setting and manualised support intervention for independence in dementia (NIDUS-Family): longer-term outcomes of a randomised controlled trial

**DOI:** 10.1192/bjo.2025.10940

**Published:** 2026-01-08

**Authors:** Melisa Yilmaz, Victoria Vickerstaff, Jessica Budgett, Julie A. Barber, Claudia Cooper

**Affiliations:** Wolfson Institute of Population Health, Queen Mary University of London, London, UK; Department of Statistical Science and Biostatistics Group, NIHR UCLH Biomedical Research Centre, London, UK; Priment Clinical Trials Unit, Research Department of Primary Care and Population Health, University College London, London, UK

**Keywords:** Dementias/neurodegenerative diseases, psychosocial interventions, goal attainment scaling, randomised controlled trial, personalised support

## Abstract

**Background:**

The new psychosocial goal-setting and manualised support intervention for independence in dementia (NIDUS-Family) is a manualised dementia care intervention.

**Aims:**

To evaluate whether goal-setting plus NIDUS-Family is more effective than the control condition (goal-setting and routine care) in supporting dyads’ (family carers and care recipients with dementia) attainment of personalised goals; and to determine participant-perceived goal relevance over 24 months.

**Method:**

We randomised dyads from community settings (2:1): to NIDUS-Family, a manualised psychological intervention tailored to goals that dyads set by selecting modules, delivered in 6–8 video call/telephone sessions over 6 months then 2–3 follow-ups monthly for 6 months; or to control. Outcomes were goal attainment scaling (GAS) (primary) at 18 and 24 months, functioning, quality of life, time until care home admission or death, carer anxiety and depression. Primary analysis, a mixed-effects model, accounted for randomisation group, study site, time, intervention arm facilitator and repeated measurements.

**Results:**

In the period 2020–2021, 204 participants were randomised to intervention and 98 to control; 164 (54.3%) and 141 (46.7%) dyads completed 18- and 24-month outcomes, respectively.

In the primary analysis, including 277 participants contributing 6-, 12-, 18- or 24-month outcomes, adjusted GAS mean differences (intervention–control) at 18 and 24 months were 11.78 (95% CI 6.64, 16.93) and 8.67 (95% CI 3.31, 14.02), respectively. Secondary outcome comparisons were not significant. The hazard ratio for dying or care home admission was 0.80 (95% CI 0.45, 1.42; intervention versus control), and 0.87 (95% CI 0.41, 1.82) and 0.59 (95% CI 0.26, 1.33) for death and care home admission, respectively. Among baseline GAS goals, carers considered 436 (78.0%) relevant at 18 months and 383 (78.5%) at 24 months.

**Conclusions:**

NIDUS-Family improved attainment of GAS goals over 2 years.

**Trial Registration Number:**

ISRCTN11425138.

Most people with dementia wish to remain living at home for as long as possible, valuing the independence, safety and familiarity that it provides.^1,2^ In the UK, approximately 700 000 unpaid family carers provide support to individuals with dementia in their homes, often expressing a willingness to do ‘whatever it takes’.^1,3^ However, without adequate support and strategies that balance the needs of people living with dementia and carers, care at home may break down, resulting in a sudden transition to a care home facility.^4^ The UK National Institute for Health and Care Excellence (NICE) guidelines stress the importance of offering psychosocial and environmental interventions to reduce stress, address behavioural and sleep disturbances with personalised strategies and provide carer support.^5^ Nevertheless, there remains a significant gap between policy and real-world implementation, leaving individuals with dementia and their carers with inadequate support.^6^

Psychosocial interventions that are written down in a manual in order that they may be delivered consistently (standardised) can be facilitated by trained, supervised staff without clinical qualifications, increasing access to evidence-based dementia care. Standardised therapies at first seem discordant with the ‘personalisation agenda’, which recognises that care is most effective if individually tailored. We co-designed, with patient and public involvement, the New Interventions for Independence in Dementia Study – Family (NIDUS-Family), a fully manualised, modular psychosocial support intervention that can be tailored to individual goals and delivered by facilitators without formal clinical training. It uses goal attainment scaling (GAS), a structured outcome measure to set goals around what is most meaningful to both carer and care recipient, with dementia pairs (henceforth dyads). NIDUS-Family significantly improved goal attainment for dyads compared with goal-setting and routine care, and was cost-effective over 1 year.^7^ Driven by our interest in understanding how intervention effects might be maintained over time and beyond the 1-year intervention period, we aimed to test whether NIDUS-Family improved dyads’ goal attainment compared with the control condition (goal-setting and routine care) for up to 2 years. Because this is the first time that GAS has been used as a trial measure beyond 1 year, we also asked participants about the relevance of baseline goals at 18- and 24-month follow-up.

## Method

### Study design and participants

The authors assert that all procedures contributing to this work comply with the ethical standards of the relevant national and institutional committees on human experimentation, and with the Helsinki Declaration of 1975 as revised in 2013. All procedures involving human subjects/patients were approved by Camden and King’s Cross Research Ethics Committee (no. 19/LO/1667) on 7 January 2020. A substantial protocol amendment (approved 19 September 2022) added 18- and 24-month follow-ups.

NIDUS-Family was a two-armed, parallel-group, single-masked, multi-site, superiority randomised controlled trial for which the protocol^8^ and 12-month primary clinical and cost-effectiveness outcomes are published.^9,10^

NIDUS-family trial participant dyads were individuals with dementia and their informal (family or friend) carers (henceforth carers). Inclusion criteria were, for the person with dementia, a documented dementia diagnosis, regardless of type or severity, and living in their own home; and for carers, being in at least weekly face-to-face or telephone contact with the person with dementia and speaking English sufficiently to complete outcome measures (where the person with dementia did not speak English, we used interpreters to engage them as far as they were able during the intervention). We excluded dyads where the person with dementia was likely to be in their last 6 months of life, either member was participating in another study or if the carer lacked the capacity to provide consent or could not identify a minimum of three appropriate GAS goals. Gender was self-reported. Written or verbal informed consent was obtained from all participants. Verbal consent was witnessed and formally recorded. Participant recruitment for the trial occurred between 30 April 2020 and 09 May 2021.

### Randomisation

The allocation process was managed via a remote web-based system provided by the the PRIMENT Clinical Trials Unit (CTU) at University College London. Randomisation was blocked and stratified by site using a 2:1 allocation ratio (intervention: control). Randomisation status was concealed from researchers collecting outcome data from carers. It was not possible to blind participants or facilitators to their assigned group.

### Interventions/procedures

Participants in the intervention arm received NIDUS-Family, a manualised intervention that can be tailored to personal goals of people living with dementia and their families. This procedure utilises components of behavioural management, carer support, psychoeducation, communication and coping skills training, enablement and environmental adaptations, with modules selected to address dyads’ selected goals.^11^ NIDUS-Family was delivered by university-employed facilitators without previous clinical training or clinical qualifications, who received manualised training from the study team focusing on clinical skills and module delivery. Facilitators delivered 6–8 manualised sessions in the first 6 months, by video or telephone. These were tailored to participant GAS goals set at baseline (see Outcome measures below). This was followed by 30 min catch-up telephone or video calls at 2 - to 3-month intervals over the next 6 months, to review progress towards goals and implementation of action plans and to troubleshoot difficulties following a standard guide. Full details of intervention facilitator training and adherence are published elsewhere.^9,10^

Participants in the control condition received usual routine care and completed goal-setting prior to randomisation. Regarding routine care, most people with dementia are diagnosed in memory services, receiving a diagnostic feedback appointment then typically with signposting to brief post-diagnostic support, including cognitive stimulation therapy groups, and anti-dementia medication if they are eligible, but provision varies widely.^12^

### Outcome measures

The trial primary outcome was family carer-rated GAS, a valid and reliable measure that is responsive to change in function in people with dementia living at home (up to 12 months) and has been adapted to dementia.^13,14^ Trained researchers collaborated with carers and individuals living with dementia to establish three to five goals (specific, measurable, achievable, realistic and time-bound (SMART)) tailored to domains such as cognition, daily living activities, self-care, mood, behaviour and mobility. Goals were designed to support the person with dementia in living well or maintaining independence at home over the following year, provided these aligned with the intervention’s scope. While goals could address carer well-being or support if these influenced the person’s functioning or quality of life, at least one goal was required to focus directly on the individual with dementia. At baseline, family carers set criteria for how to evaluate ‘performance’ on goals set, on a 5-point scale ranging from ‘much worse’ to ‘much better’ than expected. They were then reminded of these criteria at follow-ups and asked to rate goal performance. Because people had different goals and numbers of goals, a summary formula standardised the degree of goal attainment, analysed as a change score. Researchers also independently rated participant GAS attainment following completion of the main outcome battery and based on their conversations. They sought to record this independently of the carer rating by recording it ahead of the carer rating. The primary outcome in this study was family carer-rated GAS scores at 18 and 24 months. At the 18- and 24-month follow-ups, dyads were asked: ‘Is this goal still relevant to you/the person you care for?’, and the response was recorded as a yes or no. If a dyad reported that their goal was no longer relevant, they were asked why this was the case.

Information about the living status of the person with dementia was obtained at 18 and 24 months, including whether they were still alive (and if not, the date of death), whether they had moved to a care home and whether that move was permanent, the date of care home admission and the length of stay in days. To minimise assessment burden, other outcomes were completed only at 18 months. These were: the Disability Assessment for Dementia scale, a measure of functional independence (basic and instrumental activities of daily living^13^); the Dementia-Related Quality of Life (DEMQOL), a measure of quality of life of people with dementia, completed by family carers (DEMQOL-Proxy) and people with dementia if they were able (DEMQOL);^15^ the Hospital Anxiety and Depression Scale to measure family carers’ psychological morbidity;^16^ and the modified Client Service Receipt Inventory.^17^

### Statistical analysis

Intervention effects at 18 and 24 months were estimated as differences in mean family carer-rated GAS scores between allocation groups, obtained from a 3-level, mixed-effects model using all available repeated measures at 6, 12, 18 and 24 months. The model included random effects for intervention arm facilitator and the repeated measurements, with fixed effects for randomisation group, study site and time point. An interaction term between randomisation group and time point was included to estimate intervention effects at each follow-up point. We used similar models to estimate treatment effects for the secondary DEMQOL-Proxy and researcher-rated GAS outcomes. The model for DEMQOL-Proxy additionally included adjustment for baseline DEMQOL-Proxy score. Other outcome scores were summarised by group.

Time to permanent care home admission or death was calculated relative to baseline, and summarised by randomised group using Kaplan–Meier plots (see Supplementary Figs 1 and 2 available at https://doi.org/10.1192/bjo.2025.10940) for each event type, and for the composite (death or care home transition). For formal comparison of groups, the time to admission/death was analysed using a parametric shared frailty model, assuming a Weibull survival model, allowing for intervention arm facilitator clustering and adjusting for study site. Those lost or withdrawn before death or care home admission were censored at their last follow-up point. We also fitted a competing risks model, adjusted for study site, to obtain separate effect estimates for death and care home transition.

Analyses were intention to treat and included all available data, assuming that any missing values were missing at random. For our primary outcome we conducted sensitivity analyses to consider the impact of missing data on our results. We refitted our main model based on data-sets completed using multiple imputation; our imputation models included repeated-outcome measurements, sociodemographic baseline data and other variables potentially related to missingness and outcome (DEMQOL-Proxy baseline score and disability assessment for dementia baseline score), with imputations performed by study arm. A pattern-mixture approach was used to investigate missing-not-at-random scenarios. A range of *δ* values (0, 4, 8, 12) was subtracted from multiple imputation GAS scores, and regression models with fixed effects for site and treatment group were fitted for the 18- and 24- month outcomes. In addition, a worst-case sensitivity analysis was conducted where participants admitted to a care home (permanently) or who had died had their missing GAS goal values imputed with a value of −2, indicating much worse than expected.

At 18 and 24 months we asked participants, for each goal set, whether it still felt relevant. We conducted a content analysis of free-text responses to identify the main reasons why goals were no longer considered to be relevant, using an inductive content analysis approach.^18,19^

## Results

### Sample description

**Fig. 1:**
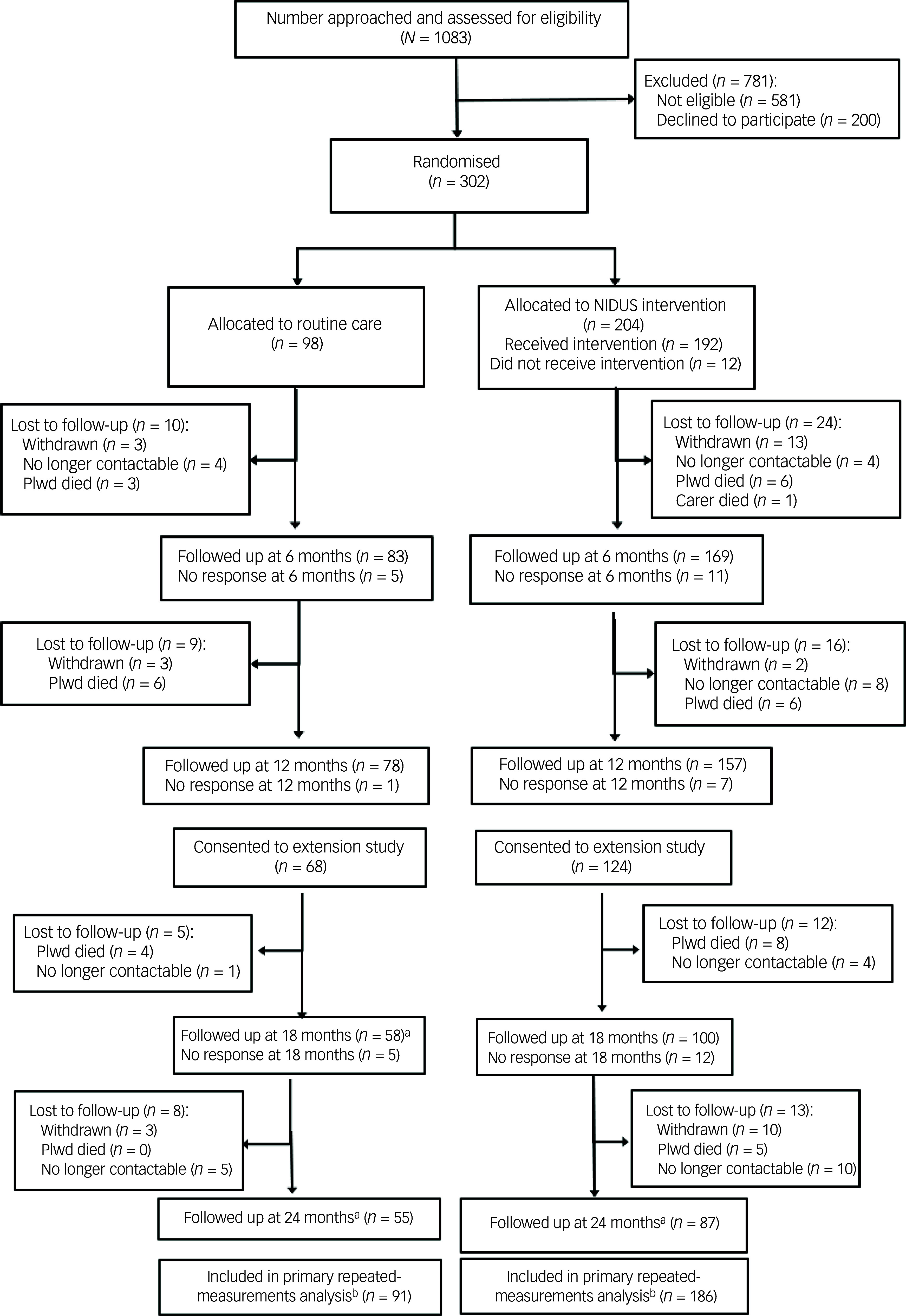
CONSORT diagram for NIDUS-Family trial. a. Numbers are those providing any data at follow-up point; in some cases this did not include GAS score (see Table 3). b. Primary analysis included all those with at least one GAS measurement during the 24-month follow-up period. CONSORT, Consolidated Standards of Reporting Trials; NIDUS-Family, New Interventions for Independence in Dementia Study – Family; GAS, goal attainment scaling; Plwd, people living with dementia.

From 302 dyads randomised, the primary outcome was available for 164 (54.3%) at 18-month follow up, and for 141 (46.7%) at 24 months. Figure 1 (Consolidated Standards of Reporting Trials diagram) illustrates the flow of participants through the study.

Tables 1 and 2 show the baseline characteristics of people living with dementia and family carers, respectively.

**Table 1:** Baseline characteristics for people living with dementia participating in the extension study, by arm

	Randomised group		
Demographics	Routine care, *n* = 68	NIDUS-Family intervention, *n* = 124	Total, *N* = 192
Age (years), mean (s.d.)	79.8 (8.5)	78.9 (8.4)	79.2 (8.4)
Ethnicity, *n* (%)			
White	56 (82.4)	98 (79.0)	154 (80.2)
White other	6 (8.8)	14 (11.3)	20 (10.4)
Mixed	2 (2.9)	0 (0)	2 (1.0)
Asian	1 (1.5)	6 (4.8)	7 (3.6)
Black	1 (1.5)	4 (3.2)	5 (2.6)
Other	2 (2.9)	2 (1.6)	4 (2.1)
First language, *n* (%)			
English	59 (86.8)	106 (85.5)	165 (85.9)
Other	9 (13.2)	18 (14.5)	27 (14.1)
Gender, *n* (%)			
Male	28 (41.2)	57 (46.0)	85 (44.3)
Female	40 (58.8)	67 (54.0)	107 (55.7)
Marital status, *n* (%)			
Married/civil partnership	41 (60.3)	68 (54.8)	109 (56.8)
Divorced	5 (7.4)	4 (3.2)	9 (4.7)
Single	0 (0)	3 (2.4)	3 (1.6)
Cohabiting	1 (1.5)	3 (2.4)	4 (2.1)
Widowed	21 (30.9)	44 (35.5)	65 (33.9)
Other	0 (0)	2 (1.6)	2 (1.0)
Education, n (%) (*n* = 189)			
Higher degree	7 (10.3)	13 (10.7)	20 (10.6)
Degree	12 (17.6)	23 (19.0)	35 (18.5)
A-level (or equivalent)	7 (10.3)	11 (9.1)	18 (9.5)
HNC/HND (or equivalent)	6 (8.8)	10 (8.3)	16 (8.5)
NVQ (or equivalent)	4 (5.9)	6 (5.0)	10 (5.3)
GCSE (or equivalent)	12 (17.6)	22 (18.2)	34 (18.0)
School Leaving Certificate	9 (13.2)	21 (17.4)	30 (15.9)
No formal qualifications	11 (16.2)	15 (12.4)	26 (13.8)
Living situation, *n* (%)			
Live alone	16 (23.5)	42 (33.9)	58 (30.2)
Live with partner/spouse	38 (55.9)	62 (50.0)	100 (52.1)
Live with children	9 (13.2)	12 (9.7)	21 (10.9)
Other	5 (7.4)	8 (6.5)	13 (6.8)
Accommodation, *n* (%)			
Council rented	4 (5.9)	8 (6.5)	12 (6.2)
Housing association rented	4 (5.9)	6 (4.8)	10 (5.2)
Private rented	3 (4.4)	5 (4.0)	8 (4.2)
Owner-occupied	55 (80.9)	94 (75.8)	149 (77.6)
Other	2 (2.9)	11 (8.9)	13 (6.8)
Dementia diagnosis, *n* (%)			
Alzheimer’s disease	32 (47.1)	57 (46.0)	89 (46.4)
Vascular dementia	6 (8.8)	14 (11.3)	20 (10.4)
Lewy body dementia	1 (1.5)	6 (4.8)	7 (3.6)
Frontotemporal dementia	2 (2.9)	3 (2.4)	5 (2.6)
Other	18 (26.5)	38 (30.6)	56 (29.2)
Unable to specify	9 (13.2)	6 (4.8)	15 (7.8)

NIDUS-Family, New Interventions for Independence in Dementia Study – Family; HNC, Higher National Certificate; HND, Higher National Diploma; NVQ, National Vocational Qualification; GCSE, General Certificate of Secondary Education.

**Table 2:** Carer characteristics, by arm, for those in the extension study

	Randomised group		
Demographics	Routine care, *n* = 68	NIDUS-Family intervention, *n* = 124	Total, *N* = 192
Carer age (years), mean (s.d.)	63.7 (10.6)	61.4 (11.5)	62.2 (11.2)
Carer ethnicity, *n* (%)			
White	54 (79.4)	93 (75.0)	147 (76.6)
White other	8 (11.8)	18 (14.5)	26 (13.5)
Mixed	1 (1.5)	0 (0)	1 (0.5)
Asian	1 (1.5)	6 (4.8)	7 (3.6)
Black	1 (1.5)	5 (4.0)	6 (3.1)
Other	3 (4.4)	2 (1.6)	5 (2.6)
Carer first language, *n* (%)			
English	63 (92.6)	115 (92.7)	178 (92.7)
Other	5 (7.4)	9 (7.3)	14 (7.3)
Carer gender, *n* (%)			
Male	25 (36.8)	29 (23.4)	54 (28.1)
Female	43 (63.2)	95 (76.6)	138 (71.9)
Carer marital status, *n* (%)			
Married/civil partnership	54 (79.4)	94 (75.8)	148 (77.1)
Divorced	4 (5.9)	1 (0.8)	5 (2.6)
Single	3 (4.4)	16 (12.9)	19 (9.9)
Cohabiting	4 (5.9)	10 (8.1)	14 (7.3)
Widowed	3 (4.4)	1 (0.8)	4 (2.1)
Other	0 (0)	2 (1.6)	2 (1.0)
Carer education, *n* (%)			
Higher degree	13 (19.1)	25 (20.2)	38 (19.8)
Degree	21 (30.9)	40 (32.3)	61 (31.8)
A level (or equivalent)	9 (13.2)	19 (15.3)	28 (14.6)
HNC/HND (or equivalent)	5 (7.4)	4 (3.2)	9 (4.7)
NVQ (or equivalent)	3 (4.4)	8 (6.5)	11 (5.7)
GSCE (or equivalent)	11 (16.2)	18 (14.5)	29 (15.1)
School Leaving Certificate	5 (7.4)	6 (4.8)	11 (5.7)
No formal qualifications	1 (1.5)	4 (3.2)	5 (2.6)
Relationship to care recipient, *n* (%)			
Spouse/partner	37 (54.4)	56 (45.2)	93 (48.4)
Child	30 (44.1)	60 (48.4)	90 (46.9)
Friend	0 (0)	1 (0.8)	1 (0.5)
Other	1 (1.5)	7 (5.6)	8 (4.2)
Carer living situation, *n* (%)			
Live alone	2 (2.9)	6 (4.8)	8 (4.2)
Live with partner/spouse	49 (72.1)	87 (70.2)	136 (70.8)
Live with flat/housemate(s)	0 (0)	1 (0.8)	1 (0.5)
Live with parent(s)	7 (10.3)	9 (7.3)	16 (8.3)
Live with children	2 (2.9)	3 (2.4)	5 (2.6)
Other	8 (11.8)	18 (14.5)	26 (13.5)
Carer accommodation, *n* (%)			
Council rented	4 (5.9)	6 (4.8)	10 (5.2)
Housing association rented	4 (5.9)	4 (3.2)	8 (4.2)
Private rented	3 (4.4)	7 (5.6)	10 (5.2)
Owner-occupied	57 (83.8)	103 (83.1)	160 (83.3)
Other	0 (0)	4 (3.2)	4 (2.1)

NIDUS-Family, New Interventions for Independence in Dementia Study – Family; HNC, Higher National Certificate; HND, Higher National Diploma; NVQ, National Vocational Qualification; GCSE, General Certificate of Secondary Education.

**Table 3:** Summary of secondary outcome scores at each follow-up point, by arm

	Baseline			6 months			12 months			18 months			24 months		
Secondary outcome	*n*	Mean	(s.d.)	*n*	Mean	(s.d.)	*n*	Mean	(s.d.)	*n*	Mean	(s.d.)	*n*	Mean	(s.d.)
Researcher GAS															
Routine care	–	–	–	86	52.4	(12.7)	84	48.7	(14.2)	59	48.6	(12.4)	52	50.0	(13.7)
NIDUS-Family intervention	–	–	–	175	59.0	(11.3)	163	58.9	(12.9)	105	60.7	(11.2)	89	58.1	(12.8)
Disability assessment															
Routine care	97	50.3	(28.3)	66	50.1	(31.7)	59	45.4	(27.8)	39	62.9	(28.4)	–	–	–
NIDUS-Family intervention	204	55.4	(27.0)	140	47.6	(29.5)	103	40.7	(28.7)	67	62.3	(28.8)	–	–	–
DEMQOL															
Routine care	36	88.2	(10.8)	14	92.7	(10.2)	8	91.1	(11.5)	8	86.9	(12.4)	–	–	–
NIDUS-Family intervention	91	90.2	(13.4)	35	88.9	(14.5)	19	91.2	(10.7)	9	88.8	(10.3)	–	–	–
DEMQOL-Proxy															
Routine care	97	88.7	(14.6)	64	94.0	(13.6)	59	90.4	(13.3)	36	91.7	(17.2)	–	–	–
NIDUS-Family intervention	204	89.9	(14.6)	141	94.1	(13.4)	103	94.8	(15.5)	65	91.3	(17.2)	–	–	–
HADS anxiety															
Routine care	98	7.1	(4.4)	65	6.8	(4.5)	59	6.7	(4.3)	39	6.9	(4.7)	–	–	–
NIDUS-Family intervention	203	6.9	(4.3)	136	6.4	(4.2)	102	6.6	(4.1)	67	7.0	(4.4)	–	–	–
HADS depression															
Routine care	98	5.4	(4.2)	65	5.4	(4.1)	59	4.8	(3.6)	39	5.3	(4.6)	–	–	–
NIDUS-Family intervention	203	5.0	(3.7)	136	4.8	(3.6)	102	4.7	(3.4)	67	5.2	(3.7)	–	–	–
HADS anxiety care (≥9)															
Routine care	98	38	(38.8)	65	23	(35.4)	59	20	(33.9)	39	14	(35.9)	–	–	–
NIDUS-Family intervention	203	71	(35.0)	136	44	(32.4)	102	28	(27.5)	67	23	(34.3)	–	–	–
HADS depression (≥9)															
Routine care	98	28	(28.6)	65	13	(20.0)	59	13	(22.0)	39	9	(23.1)	–	–	–
NIDUS-Family intervention	203	24	(11.8)	136	22	(16.2)	102	15	(14.7)	67	11	(16.4)	–	–	–

GAS, goal attainment scaling; NIDUS-Family, New Interventions for Independence in Dementia Study – Family; DEMQOL, Dementia-Related Quality of Life; DEMQOL-Proxy, DEMQOL completed by family carers; HADS, Hospital Anxiety and Depression Scale.

Supplementary Tables 1–4 show the characteristics of people living with dementia and family carers who did and did not take part in the extension study. Compared with those who did not take part, people with dementia and carers who took part in the extension study were slightly younger, but otherwise characteristics were very similar.

### Comparison of primary outcome between arms

**Fig. 2:**
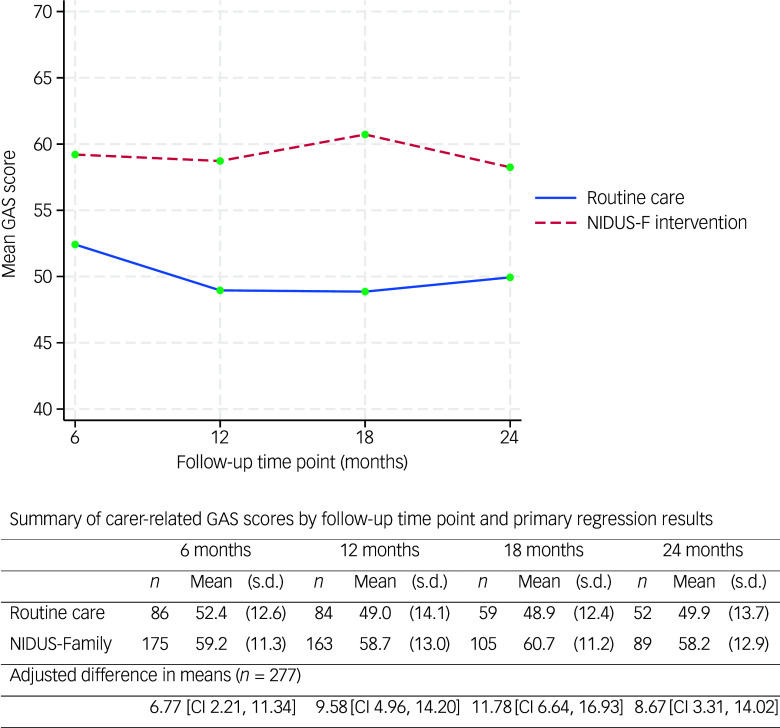
The primary outcome (carer-rated GAS scores) over 24 months, by arm. GAS, goal attainment scaling; NIDUS-F/NIDUS-Family, New Interventions for Independence in Dementia Study – Family.

The mean family carer-rated GAS score was significantly higher in the intervention than the control arm at all time points (Fig. 2).

We included 277 people living with dementia and who had at least one primary outcome measurement recorded (at 6, 12, 18 or 24 months) in our mixed-effects model. In this model, the adjusted difference in means (NIDUS control group) at 18-month follow-up was 11.78 (95% CI 6.64, 16.93), and 8.67 (95% CI 3.31, 14.02) at 24 months. These estimates were very similar in multiple imputation and worse-case scenario sensitivity analyses. Supplementary Table 5 shows findings from the pattern-mixture approach that we used to investigate missing-not-at-random scenarios. For 18-month outcomes the intervention effect remained significant with all assumptions, while for 24-month outcomes it remained significant for all but some of the most extreme scenarios.

### Comparison of secondary outcomes

Table 3 summarises secondary outcome scores at all time points.

Researcher-rated GAS mean scores were higher in the NIDUS-Family intervention compared with the control arm across all time points. Differences in means between groups were significant in adjusted analyses: 11.99 (95% CI 8.09, 15.90) at 18 months and 8.94 (95% CI 4.81, 13.06) at 24 months. The adjusted treatment effect estimates for DEMQOL-Proxy were not statistically significant: adjusted difference in means of 2.36 (95% CI −1.59, 6.30) at 18 months. Treatment effect estimates for these outcomes are reported in Supplementary Table 6. Other scores were similar across both arms (Table 3).

### Care home admission and mortality

By 24 months, 15 (15.3%) control arm participants and 25 (12.2%) intervention arm participants were know to have moved to a care home, while 13 (13.3%) control arm and 25 (12.3%) intervention arm participants were known to have died (Supplementary Table 7). The hazard ratio for dying or moving to a care home was 0.80 (95% CI 0.45, 1.42) for the NIDUS-Family intervention compared with routine care, and sub-hazard ratios for death and care home admission were 0.87 (95% CI 0.41, 1.82) and 0.59 (95% CI 0.26, 1.33), respectively. The latter indicates a reduction in the hazard of permanent care home admission of 41% in the intervention relative to the control group, although this is not statistically significant.

### Goal relevance

The 164 carers rating GAS goals at 18 months evaluated performance on 559 goals, of which 436 (78.0%) were reported by carers as still feeling relevant. The 141 carers rating GAS goals at 24 months evaluated performance on 488 goals, of which 383 (78.5%) still felt relevant.

The reasons why goals no longer felt relevant included: the goals having being achieved (7 goals at 18 months, 12 goals at 24 months); the person with dementia having died (30 goals at 18 months, 4 goals at 24 months); moving to a care home (15 goals at 18 months, 9 goals at 24 months); the goals becoming unrealistic due to a decline in health of the person living with dementia (12 goals at 18 months, 30 goals at 24 months); the goals no longer aligning with the dyad’s priorities, because they had accepted the person’s current functional state and shifted focus (5 goals at 18 months, 14 goals at 24 months); no observable change (4 goals at 18 months, 1 goal at 24 months); and external circumstances or stressors preventing carers progressing towards the goals (3 goals at 24 months), as detailed in Supplementary Table 8. Supplementary Table 9 provides examples for each of these reasons for goals no longer feeling relevant.

## Discussion

We have previously reported that both goal-setting and NIDUS-Family improved dyads’ goal attainment compared with goal-setting and routine care, and was cost-effective over 1 year.^9,10^ In the current study, we report that these measures continued to improve goal attainment for up to 2 years.

The reduction in the rate of care home admission that we found in the intervention group, although not statistically significant, was of a similar magnitude to that in previous findings. In a meta-analysis (*N* = 9053), the odds ratio for the association of likelihood of institutionalisation with interventions designed to improve home support versus control was 0.66 (95% CI 0.43, 0.99). In two US studies that informed the NIDUS-Family theoretical development,^20^ home support interventions for family carers and people living with dementia also delayed institutionalisation by around one third (hazard ratio 0.65 [95% CI 0.45, 0.94]^21^ and 0.63 [0.42, 0.94], respectively^22^). A commonality of these interventions was a goal-focused approach to supporting care recipients with dementia and family carers. This strategy, of identifying dyads’ priorities when planning how to enable continued care at home, may be critical to extending time lived at home. There is little evidence that carer psychological stress alone increases institutionalisation,^23^ or that interventions focused primarily on reducing carer stress prevent it.^24^

This is, to our knowledge, the first use of GAS as a trial outcome measure over a period greater than 1 year, with most previous studies using it over 3 months or less.^25^ It is encouraging that nearly 8 in 10 goals set by participants felt relevant for up to 2 years, suggesting that GAS can be a useful outcome over this longer period. Average proxy-rated quality of life scores in the intervention versus control arm were not statistically significant, and of a similar magnitude to those recorded at 12 months in the NIDUS-Family trial; we previously noted that this may be a clinically important effect size^26^ that we lacked the power to detect. Alternatively, goal attainment measures might be more sensitive to clinically important change, or could be measuring distinct concepts to the secondary, generic outcomes, which tend to focus on impairment as opposed to capturing strengths and well-being. We asked dyads to set goals towards living as long and as well as possible at home; many people with dementia and family carers consider remaining at home a priority, even where it brings challenges.

There are some limitations to our research. We asked carers to set goals, to which people living with dementia contributed to the extent they were able. We cannot independently verify how goals reflected the probable wishes of people with dementia who lacked capacity. Unsurprisingly, both researcher- and carer-rated GAS scores were very similar, probably because the former were based on carers’ reports regarding goal attainment, and thus the extent to which the researchers’ rating provided an alternate perspective is limited.

Because the evidence base for the current intervention is limited to included populations, it is not directly relevant to carers unable to understand written English or people with dementia and without a regular carer. We are currently exploring how to extend support to these groups through developing a course for home carers called NIDUS-Professional,^27^ and in developing the materials in other languages.

Participants originally consented to complete outcomes for 1 year and, while most agreed to take part in this extension study, response rates were lower than for previous time points. We maximised participation by minimising assessment burden, and thus we had very limited measures at 24 months. While sensitivity and multiple imputation models have evaluated the potential impact of missing data, they cannot fully mitigate the likelihood that the half of clients followed up to the end of the study differs from that who were not: for example, completers may have had less cognitive and functional decline than non-completers.

Because NIDUS-Family is delivered by professionals without formal clinical training, it can extend the workforce capable of delivering evidence-based dementia care. Nonetheless, sufficient workforce resources are needed, and limited resources were noted to be a significant implementation barrier to wider delivery across health and social care sectors in our recent pre-implementation study.^28^ Major cuts in social care budgets increase the risk of high-cost care home admissions, which older people do not want.^29^ In England, inadequate adult social care availability (for care home placements and home care support) has led to more requests for help being turned down in the period 2023–2024, delaying hospital discharge and reducing the capacity of people to live as independently as possible in their own homes.^30^ Limited dementia skills and training within the health and social care workforce represent a further barrier to implementation.^31,32^

The NIDUS-Family dementia care intervention improved personalised goal attainment, on GAS goals that remained relevant for most dyads, over 2 years. Most people with dementia prefer to remain in their own homes, and this is best enabled through the provision of good-quality, personalised community health and social care. NIDUS-Family is a potentially important tool to enable this, with materials available free of charge (gracedementiacare.co.uk) and deliverable following appropriate training and with supervision.

## Supporting information

Yilmaz et al. supplementary material Yilmaz et al. supplementary material

## Data Availability

The data collected for the study, including the statistical analysis plan, deidentified participant data and a data dictionary defining each field in the set will be made available to others on receipt by Priment Clinical Trials Unit (CTU) (priment@ucl.ac.uk) following reasonable request, at any date following publication of this paper. All requests will be reviewed by Priment CTU in line with Priment CTU guidance on sharing data and anonymising data. This process is in place to ensure that the request is reasonable and that the data-set suitably anonymised. The study protocol is available via open access. Intervention materials are available without cost, subject to an Attribution-NonCommercial-NoDerivatives licence held by C.C., chief investigator.

## Supplementary material

The supplementary material is available online at https://doi.org/10.1192/bjo.2025.10940
